# Genetic testing and serological screening for SARS-CoV-2 infection in a COVID-19 outbreak in a nursing facility in Japan

**DOI:** 10.1186/s12879-021-05972-5

**Published:** 2021-03-15

**Authors:** Yong Chong, Naoki Tani, Hideyuki Ikematsu, Nobuto Terazawa, Hitoshi Nakashima, Nobuyuki Shimono, Koichi Akashi, Yosuke Tanaka

**Affiliations:** 1 Medicine and Biosystemic Science, Kyushu University Graduate School of Medical Sciences (The First Department of Internal Medicine), 3-1-1 Maidashi, Higashi-Ku, Fukuoka, 812-8582 Japan; 2 Japan Physicians Association, Tokyo, Japan; 3 Medical Corporation SOUSEIKAI, Kanenokuma Hospital, Fukuoka, Japan; 4 Center for the Study of Global Infection, Kyushu University Hospital, Fukuoka, Japan

**Keywords:** PCR testing, Antibody testing, COVID-19 outbreak, Nursing facility, SARS-CoV-2

## Abstract

**Background:**

The Pandemic of coronavirus disease (COVID-19), caused by severe acute respiratory syndrome coronavirus 2 (SARS-CoV-2), has critically impacted the spread of infection within nursing facilities. We evaluated the usefulness of genetic and serological tests conducted during a COVID-19 outbreak in a nursing facility in Japan.

**Methods:**

After the first identification of SARS-CoV-2 infection, a comprehensive, facility- and/or unit-wide PCR testing from nasopharyngeal swabs was repeatedly performed in a three-unit facility including 99 residents with dementia and 53 healthcare personnel. Additionally, PCR testing was conducted separately for residents and staff with fever of ≥37.5 °C. Facility-wide serological testing, including rapid kit testing and quantitative assay, was conducted twice over 1 month apart.

**Results:**

A total of 322 PCR and 257 antibody tests were performed. 37 (24.3%) of the 152 individuals (25/99 residents, 25.3%; 12/53 staff, 22.6%) were identified as PCR-positive. Seven residents died with a mortality of 7.1% (7/99). Among the 37 individuals, 10 (27.0%) were asymptomatic at the time of testing. PCR positivity was concentrated on one unit (Unit 1) (20/30 residents, 66.7%; 9/14 staff, 64.3%). The other units showed a limited spread of infection. In unit-wide and separate tests, PCR positivity detection was highly prevalent (22.9 and 44.4%, respectively) in Unit 1, compared with that in the other units. Serological testing identified two additional infected residents with a negative PCR result and showed that no staff was newly identified as infected.

**Conclusions:**

Thorough PCR testing, in combination with comprehensive and separate tests, is critical for managing COVID-19 outbreaks in nursing facilities, particularly, in units considered an epicenter. Serological testing is also beneficial for tracing contacts, confirming the number of infected individuals, and authorizing the termination of the outbreak.

**Supplementary Information:**

The online version contains supplementary material available at 10.1186/s12879-021-05972-5.

## Background

The coronavirus disease (COVID-19), caused by severe acute respiratory syndrome coronavirus 2 (SARS-CoV-2), has spread worldwide, and the World Health Organization declared the pandemic state on March 11, 2020. In Japan, the number of infected people started to increase from the end of February, and the infection rapidly spread from late March, reaching the peak of the first wave in mid-April. COVID-19 outbreaks within hospitals have been regarded as a critical concern and attracted great attention [1]. Contrastingly, not much attention has been paid to such outbreaks in long-term elderly care facilities. In some countries, nursing facility residents have been reported to account for a high percentage of deaths due to COVID-19, and infection control at these facilities has been stressed as an urgent issue because the control measures are greatly lacking in the nursing facilities [2, 3]. In Japan, COVID-19 outbreaks in nursing facilities have not been fully investigated, and the actual status is unclear [4].

Comprehensive polymerase chain reaction (PCR) testing during the spread of SARS-CoV-2 in nursing facilities has been reported [5–7]. This facility-wide PCR testing showed a high prevalence of infection at approximately 40–80% and the contribution to rapid transmission by asymptomatic infected residents at the time of testing. These reports suggest the importance of prompt, comprehensive PCR testing to detect residents with no symptoms in nursing facilities.

We recently experienced a COVID-19 outbreak in a 100-bed long-term care facility in a local city of 1.5 million individuals in Japan. The nursing facility, mostly occupied by residents with dementia, experienced the spread of SARS-CoV-2 in April. There was a serious concern that the virus had rapidly spread across the facility because most residents had difficulties in cooperating with basic infection control measures. In this facility, from the beginning of the outbreak, multiple PCR tests were performed separately for residents and staff with fever, as well as repeated facility/unit-wide testing. Additionally, multiple facility-wide antibody testing was planned and implemented. Our experience could provide useful information on future control measures for COVID-19 outbreaks in nursing facilities.

## Methods

### Study definition

Clinical data on symptoms were collected from the medical records kept at the facility. There were few records regarding the subjective and objective symptoms characteristic of COVID-19. This was probably because most residents were not able to communicate their subjective symptoms, such as shortness of breath, which is typical for COVID-19, owing to severe dementia, and the detailed recording of objective symptoms depended on the nursing staff. Therefore, only fever and cough were defined as symptoms related to COVID-19 in terms of data reliability.

Herein, two types of PCR testing, comprehensive (facility/unit-wide) and separate tests, were performed. In comprehensive PCR testing, residents and staff with a positive result were classified as symptomatic if they had a temperature of ≥37.5 °C (fever) and/or cough within 14 days prior to testing. PCR-positive residents and staff were classified as presymptomatic if they were not classified as symptomatic at the time of testing and developed fever and/or cough within 7 days after testing. PCR-positive residents and staff were classified as asymptomatic if they were not considered symptomatic or presymptomatic. PCR testing was separately conducted when residents and staff had fever, regardless of coughing, resulting in being classified as symptomatic at the time of PCR positivity. PCR testing was performed in individuals with a fever with ≥37.5 °C according to the recommendations of the Ministry of Health, Labor and Welfare of Japan.

### Laboratory testing

Nasal or pharyngeal swab specimens for the PCR assay were collected in the facility. One-step real-time reverse transcriptase-PCR was performed by the Fukuoka City Institute of Health and Environment, according to the Manual for the Detection of Pathogen 2019-nCoV by the National Institute of Infectious Diseases [8]. Cycle threshold (Ct) values below 40 cycles were determined as positive for SARS-CoV-2.

Serological testing was performed using the serum samples collected for comprehensive testing and those remaining from other biochemical tests. Rapid antibody kit testing was conducted as previously described [9]. The result of IgM antibody detection was not used in this report because of the low ability to detect IgM, which was obtained in our preliminary research [9]. Antibody testing for PCR-positive residents and staff was performed basically after 14 days or more. In the case of positive PCR and negative IgG results, additional antibody testing was performed.

The quantitative levels of IgG antibodies for SARS-CoV-2 were examined using the same serum samples used for the rapid antibody testing. A chemiluminescent microparticle immunoassay, based on the nucleocapsid antigen of SARS-CoV-2 (SARS-CoV-2 IgG reagent kit, Abbott Laboratories Co., Ltd., Park, IL, USA), was used and performed on an ARCHITECT i2000SR automated analyzer, according to the manufacturer’s instructions. Calculated signal-to-cutoff values of ≥1.4 indicated positive detection of anti-SARS-CoV-2 IgG antibodies.

### Statistical analyses

Categorical variables were analyzed using Fisher’s exact test. Continuous variables were compared using the Wilcoxon rank-sum test. The association between two variables was analyzed using Spearman’s rank correlation test. *P* values of < 0.05 were considered statistically significant. All statistical analyses were performed using JMP Pro, version 14 (SAS Institute, Inc., Cary, NC, USA).

## Results

### Outbreak description

**Fig. 1:**
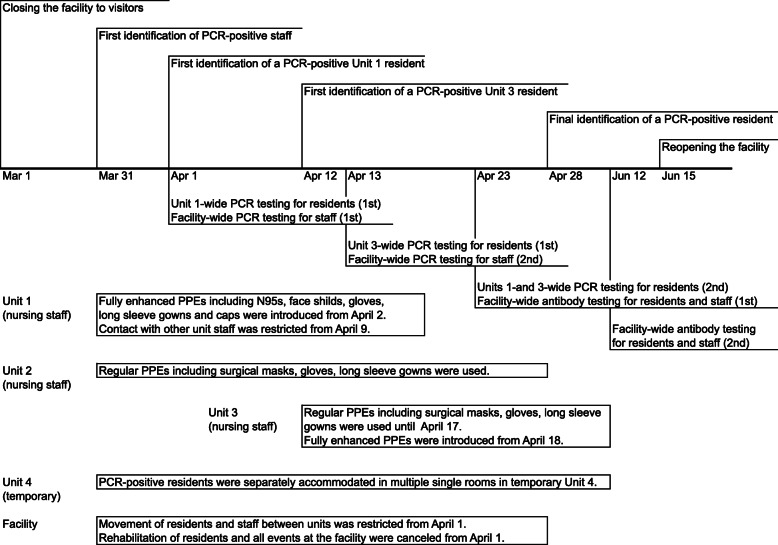
COVID-19 outbreak timeline at the facility. Implementation of genetic and serological tests based on the identification of PCR-positive residents and staff is shown. Major infection prevention and control measures implemented in the facility are shown below the timeline. Out-of-facility contact with staff in Unit 1 and staff from other units were not completely restricted before April 8. PPE, personal protective equipment; COVID-19, coronavirus disease

In Fukuoka City, Japan, the first case of COVID-19 was reported in late February, 2020, and the number of SARS-CoV-2-infected cases gradually increased in March; the first wave of the COVID-19 epidemic peaked in April. The facility reported herein is a 100-bed nursing facility divided into three units with long-term residents; it included 99 residents and 53 staff (full-time healthcare personnel) as of April 1 (Table 1). Most residents had developed advanced dementia (Table 1). All visitors were restricted to the facility from the beginning of March (Fig. 1). On March 31, a nursing staff who had worked in Unit 1 developed a fever and underwent PCR testing for SARS-CoV-2 in another hospital, resulting in the first identification of COVID-19 in the facility on April 1. Following this result, the administrative leader at the facility decided to conduct PCR testing for all residents in Unit 1 and comprehensive testing for all staff, under the permission of the Heath and Welfare Center in Fukuoka City; comprehensive and separate PCR tests were repeated during the outbreak in the facility. After the identification of PCR-positive residents and staff, infection control measures in each unit were enhanced (Fig. 1). The PCR-positive residents were accommodated and isolated in a room in Unit 4 that was not usually used immediately after identification. Subsequently, residents with severe conditions were transferred to hospitals, and other residents were treated in the facility. The PCR-positive staff were hospitalized or remained at home immediately after determining the result. The authors, belonging to Kyushu University, were requested by the administrative leader to perform serological testing in the facility, resulting in the initiation of antibody testing on April 23. The final PCR-positive resident in the facility was identified on April 28. All previously PCR-positive residents were retested and were PCR-negative by June 1. Subsequently, a second facility-wide antibody testing was implemented to confirm the termination of the COVID-19 outbreak in the facility. The facility was reopened to new residents and visitors on June 15.

**Table 1:** Demographic characteristics in residents of the facility

Characteristic	Unit 1	Unit 2	Unit 3	Total
Total number of residents^a^	30	35	34	99
Gender, male	13 (43.3)	13 (37.1)	10 (29.4)	36 (36.4)
Age, mean years ± SD (range)	84.0 ± 7.8 (69–104)	84.9 ± 7.0 (66–99)	85.2 ± 9.1 (60–105)	84.7 ± 8.0 (60–105)
ADL				
ADL scores (0–6)^b^, mean scores	2.1	1.9	1.5	1.8
Independent walking	13 (43.3)	9 (25.7)	12 (35.3)	34 (34.3)
Chronic underlying disease				
Dementia	28 (93.3)	33 (94.3)	34 (100.0)	95 (96.0)
HDS-R (0–30)^c^, mean scores	6.7	8.3	4.3	6.3
Wandering behavior due to dementia	9 (30.0)	5 (14.3)	7 (20.6)	21 (21.2)
Hypertension	9 (30.0)	12 (34.3)	9 (26.5)	30 (30.3)
Cardiac disease	8 (26.7)	8 (22.9)	3 (8.8)	19 (19.2)
Chronic pulmonary disease	2 (6.7)	3 (8.6)	3 (8.8)	8 (8.1)
Renal disease	1 (3.3)	3 (8.6)	2 (5.9)	6 (6.1)
Cerebrovascular disease	5 (16.7)	11 (31.4)	10 (29.4)	26 (26.3)
Hepatic disease	2 (6.7)	3 (8.6)	4 (11.8)	9 (9.1)
Diabetes	2 (6.7)	6 (17.1)	8 (23.5)	16 (16.2)
Hyperlipidemia	2 (6.7)	2 (5.7)	5 (14.7)	9 (9.1)
Cancer	2 (6.7)	2 (5.7)	2 (5.9)	6 (6.1)

Data are no. (%) of residents, unless indicated otherwise

^a^ Data include all residents who were present in the facility on April 1, 2020

^b^ ADL scores indicate physical self-maintenance scale (PSMS) with a total maximum score of 6

^c^ HDS-R is a screening test for age-associated dementia. Dementia was defined as a score of HDS-R ≤ 20 (total maximum score of 30)

ADL, activities of daily living; HDS-R, the revised Hasegawa’s dementia scale

### PCR testing

**Fig. 2:**
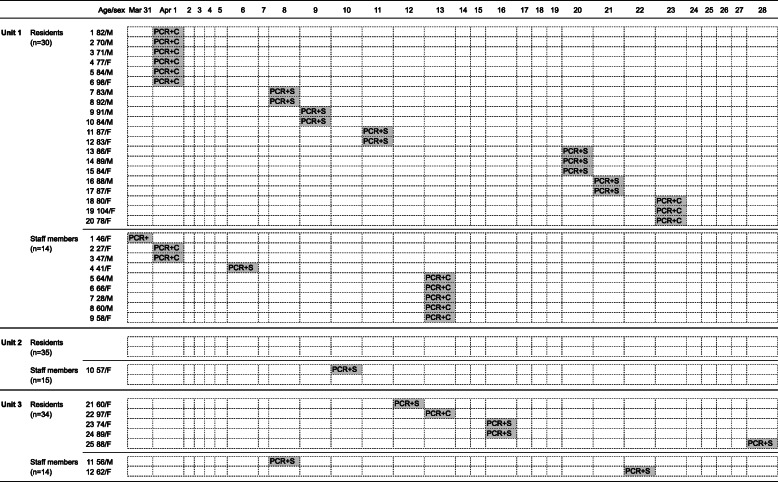
Detection timeline of residents and staff with positive PCR results for SARS-CoV-2. The number of residents and staff in each unit represents the data as of April 1, 2020. The PCR + C and PCR + S indicate PCR positivity in comprehensive (facility/unit-wide) and separate PCR testing, respectively. Staff no. 1 in Unit 1 was identified as PCR-positive in another hospital on March 31, 2020. SARS-CoV-2, severe acute respiratory syndrome coronavirus 2

A total of 283 real-time PCR tests were performed between April 1 and 28, finally reaching 322 tests including additional 39 tests performed by June 15. From March 31 to April 28, 37 of the 152 individuals (24.3%) in the facility (25/99 residents, 25.3%; 12/53 staff, 22.6%) were identified as PCR-positive for SARS-CoV-2 (Fig. 2). Seven residents, including four in Unit 1 and three in Unit 3, died after identification, resulting in a mortality of 7.1% (7/99). No staff died in the facility. PCR positivity detection was most pronounced in Unit 1 (20/30 residents, 66.7%; 9/14 staff, 64.3%), followed by Unit 3 (5/34 residents, 14.7%; 2/14 staff, 14.3%). Only one staff member was identified as PCR-positive in Unit 2. This test was possibly false-positive based on the subsequent negative results on serial antibody testing.

Table 2 shows the detection rate of real-time PCR positivity for SARS-CoV-2 in facility/unit-wide and other separate tests. PCR positivity in the comprehensive testing was highly prevalent in Unit 1 (16/70 PCR tests in residents and staff, 22.9%), compared with that in the other units. Additionally, a detection rate of 44.4% (12/27 PCR tests in residents and staff) in Unit 1 was obtained when residents and staff with fever were separately tested. The frequency of febrile episodes during the outbreak in Unit 1 was not different between the PCR-positive and -negative residents (Table S1).

**Table 2:** Facility/Unit-wide and separate PCR tests during the COVID-19 outbreak in the facility

	Positive results of PCR assay for SARS-CoV-2 (%)					
	Facility/Unit-wide testing^a^				Separate testing^b^	
	April 1–2	April 13–14	April 23–24	Subtotal	April 1–28	Total
Unit 1						
Residents (*n* = 30)	6/30 (20.0)	N/A	3/13 (23.1)	9/43 (20.9)	11/24 (45.8)	20/67 (29.9)
Nursing staff members (*n* = 14)	2/13 (15.4)	5/10 (50.0)	0/4 (0.0)	7/27 (25.9)	1/3 (33.3)	8/30 (26.7)
Unit 2						
Residents (*n* = 35)	N/A	N/A	N/A	N/A	0/17 (0.0)	0/17 (0.0)
Nursing staff members (*n* = 15)	0/15 (0.0)	0/14 (0.0)	N/A	0/29 (0.0)	1/5 (20.0)	1/34 (2.9)
Unit 3						
Residents (*n* = 34)	N/A	1/33 (3.0)	0/29 (0.0)	1/62 (1.6)	4/10 (40.0)	5/72 (6.9)
Nursing staff members (n = 14)	0/14 (0.0)	0/13 (0.0)	0/11 (0.0)	0/38 (0.0)	2/4 (50.0)	2/42 (4.8)
Other staff members (*n* = 10)	0/10 (0.0)	0/10 (0.0)	N/A	0/20 (0.0)	0/1 (0.0)	0/21 (0.0)
Total	8/82 (9.8)	6/80 (7.5)	3/57 (5.3)	17/219 (7.8)	19/64 (29.7)	36/283 (12.7)^c^

^a^ Facility/unit-wide PCR testing on April 13–14 and 23–24 was performed for the remaining residents and staff of the unit, excluding PCR-positive

individuals up to each time point

^b^ The timeline of PCR positivity in the separate PCR testing is shown in Fig. 2

^c^ The number of residents and staff with a positive PCR result does not include a case of staff 1 in Unit 1 who was identified as PCR-positive in another hospital on March 31, 2020

COVID-19, coronavirus disease 2019; SARS-CoV-2, severe acute respiratory syndrome coronavirus 2; N/A, not available

The mode of symptom onset was examined in 17 residents and staff who were identified using facility/unit-wide PCR testing (Table S2). Among them, 10 (58.8%) did not develop any symptoms at the time of testing (presymptomatic, *n* = 6; asymptomatic, *n* = 4). The real-time PCR Ct values for these 10 individuals with no symptoms at the time of testing were similar to those for the symptomatic individuals (Fig. S1).

### Serological testing

A total of 257 rapid antibody kit tests were performed by June 15. A part of serum samples used for these tests was applied to antibody quantification assay (Table S3). Among the 37 PCR-positive residents and staff, serum samples from the 33 individuals were obtained. Of these 33 individuals, 31 were positive for IgG antibodies in both kit and quantification tests (Fig. S2a). One resident who was negative for IgG in the kit testing showed IgG positivity in the quantification assay (Fig. S2a, resident no. 25 in Table S3). One staff showed IgG negativity between the kit and quantification tests, suggesting a possible false-positive result in the PCR testing with a Ct value of 19.6 (Fig. S2a, staff no. 10 in Table S3).

**Fig. 3:**
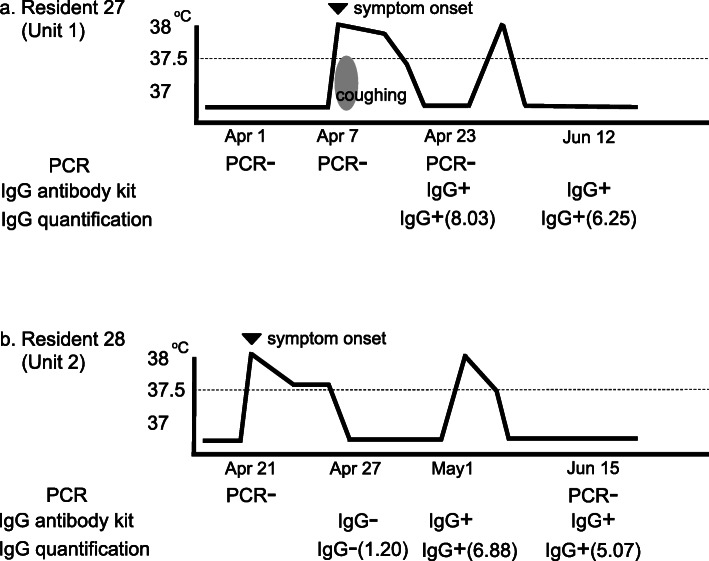
Clinical course of residents with PCR-negative and IgG-positive results for SARS-CoV-2. The serum sample obtained from resident no. 28 on May 1 was the one remaining from biochemical testing. The calculated signal-to-cutoff values of ≥1.4 indicate positive detection of anti-SARS-CoV-2 IgG antibodies. SARS-CoV-2, severe acute respiratory syndrome coronavirus 2

No staff had positive IgG testing results in both the first and second facility-wide tests (Table 3). In the first testing, three residents showed IgG positivity. One resident in Unit 2 had a negative IgG result in the quantification assay, resulting in a false-positive result in the kit testing (resident no. 26 in Table S3). Of the two remaining residents in Unit 1, one showed both positive PCR and IgG results, as of April 23 (resident no. 20 in Table S3). Another resident had negative PCR and positive IgG results, as of April 23. (resident no. 27 in Fig. 3a). In the second testing, one resident in Unit 2 was newly identified as IgG-positive (Table 3). This resident became seropositive for IgG after an initial negative status, although PCR positivity had never been detected by June 15 (resident no. 28 in Fig. 3b). Thus, serological testing most likely identified two residents infected with SARS-CoV-2 who could not be detected in the series of PCR testing. Finally, as of June 15, serological testing showed that no individuals in the facility were newly identified as infected since April 29.

**Table 3:** Facility-wide rapid antibody kit testing performed in the facility

	Positive results of IgG for SARS-CoV-2		
	Facility-wide testing		
	April 23-27^a^	June 12-15^b^	Total
Unit 1			
Residents (*n* = 13)	2/13^c^	1/10^c^	3/23
Nursing staff members (*n* = 4)	0/4	0/4	0/8
Unit 2			
Residents (*n* = 34)	1/34^c^	2/32^c^	3/66
Nursing staff members (*n* = 14)	0/14	0/14	0/28
Unit 3			
Residents (*n* = 29)	0/29	0/28	0/57
Nursing staff members (*n* = 12)	0/12	0/12	0/24
Other staff members (*n* = 10)	0/10	0/8	0/18
Total	3/116	3/108	6/224

^a^The testing was performed for PCR-negative residents and staff who were present in the facility as of April 22, 2020.

^b^ The testing was performed for PCR-negative residents and staff who were present in the facility as of June 11, 2020.

^c^ These include repeated IgG-positive results in the same resident.

SARS-CoV-2, severe acute respiratory syndrome coronavirus 2

## Discussion

Several circumstances can be identified with a presymptomatic or an asymptomatic status with positive PCR results at the time of comprehensive testing during outbreaks in nursing facilities [6, 7]. In this study, a high frequency of PCR positivity detection in comprehensive testing was observed in Unit 1, which became the center of the outbreak in the facility, leading to the identification of asymptomatic individuals at the time of testing. We re-recognized the usefulness of facility/unit-wide PCR testing during the COVID-19 outbreak in our nursing facility. In Unit 1, PCR positivity was also detected at a high frequency when separate testing was performed. Identifying PCR-positive individuals with fever as early as possible by conducting separate testing is important to prevent the spread of SARS-CoV-2. The PCR-negative residents in Unit 1 had febrile episodes at the same level during the outbreak in the unit. This implies that there are limits to infection control measures based only on symptoms. It is also particularly difficult to isolate all residents with fever. The “test-based strategy” is stressed as an infection prevention and control method for COVID-19 in nursing facilities [10]. Performing mass PCR testing in combination with comprehensive and separate tests as promptly as possible is effective for controlling COVID-19 outbreaks in nursing facilities.

Resident no. 20 in Unit 1 was identified on the basis of both PCR and IgG positivities on the same collection day (April 23). In our preliminary research using the same antibody kit applied herein, IgG antibodies were detected from day 7 after symptom onset, and the detection rate reached 100% on day 13 or later [9]. Accordingly, this resident in Unit 1 might have spread the virus for at least 1 week. The clinical courses of residents nos. 27 and 28 likely suggested that a false-negative result was obtained from the PCR tests performed on April 7 and 21 (presumed symptom onset days), respectively (Fig. 3). The false-negative rate of PCR testing for SARS-CoV-2 has been reported to be the highest on the day of symptom onset [11]. Considering the subsequent persistence of fever in these residents, multiple tests might have resulted in PCR positivity. Resident no. 27, similar to resident no. 20, might have contributed to the spread of SARS-CoV-2 in Unit 1 for approximately 2 weeks. Further thorough measures, such as weekly unit-wide PCR testing, could contribute to the prompt detection of infected residents in units that are considered hot spots for COVID-19 in nursing facilities.

IgG antibody production was observed in all residents and staff infected with SARS-CoV-2 in the facility, except for four residents in whom serum samples were not obtained and one staff with a possible false-positive PCR result. IgG antibodies for SARS-CoV-2 were found to be produced regardless of age (Fig. S2b). Additionally, IgG production seemed to persist for at least 2 months after the infection (Fig. S2c). Thus, serological testing can effectively be used to demonstrate SARS-CoV-2 infection in elderly nursing facilities. Rapid and simple antibody kit testing could be useful for practical outbreak settings. Serological testing is not recommended for the diagnosis of an active SARS-CoV-2 infection [10], and has been rarely reported in COVID-19 outbreaks in nursing facilities. Our investigation suggests that serological testing could be useful for tracing close contacts, confirming the number of infected residents and staff, and authorizing the termination of outbreaks in nursing facilities.

This study has some limitations. First, the description of symptoms characteristic of COVID-19 is scarce. This could not be avoided because of the difficulty in accurately extracting symptoms as described in the methods section. Symptomatic status misclassification for some asymptomatic individuals might have occurred, aside from presymptomatic individuals who were all subsequently in a febrile status. Second, although the reliability of the rapid antibody kit used was verified using a quantification assay, the accuracy of the assay has not been guaranteed. It was previously reported that the sensitivity and specificity were 100 and 99.6%, respectively, for the performance of this assay [12, 13]. Therefore, the reliability of the quantification assay used in this study is presumed to be extremely high.

## Conclusions

Mass PCR testing, in combination with comprehensive and separate tests, is critical for managing COVID-19 outbreaks in nursing facilities. Thorough PCR testing should be conducted for prompt detection of infected individuals, especially in facility units considered an outbreak epicenter. Serological testing is also useful for tracing close contacts, confirming the number of infected individuals and authorizing the termination of outbreaks. In the current situation in which there is no vaccination or chemoprophylaxis for SARS-CoV-2 infection, the COVID-19 outbreaks in nursing facilities could cause catastrophic consequences. We believe that our experience could be of significant use to many healthcare professionals for future infection control measures.

## Supplementary Information

**Additional file 1: Supplementary Table S1.** Demographic characteristics and febrile episodes in Unit 1 residents based on PCR positivity.

**Additional file 2: Supplementary Table S2.** Symptomatic status of residents and staff with positive PCR results in Facility/Unit-wide testing

**Additional file 3: Supplementary Table S3**. Result list of IgG antibody quantification assay.

**Additional file 4: Supplementary Figure S1.** Cycle threshold values for residents and staff with a real-time PCR-positive result for SARS-CoV-2 according to their symptom status. Cycle threshold values for the first positive PCR results of 37 residents and staff were compared between two groups classified based on the symptomatic status at the time of testing (symptomatic vs. presymptomatic/asymptomatic) The symptomatic status is defined in the methods section. The white circles indicate data of individuals with asymptomatic status. The horizontal solid bars indicate the median values. SARS-CoV-2, severe acute respiratory syndrome coronavirus 2

**Additional file 5: Supplementary Figure S2.** IgG quantification assay in residents and staff with PCR-positive results for SARS-CoV-2. **a.** Relationship of IgG positivity between rapid kit and quantification tests in 33 PCR-positive residents and staff. The gray circle indicates resident no. 25 from Table S3. The white circle indicates staff no. 10 from Table S3. **b, c.** Association of age (b) and duration after the first PCR positivity (c) with quantitative IgG values in 32 PCR-positive and quantitative IgG-positive residents and staff. The horizontal dotted lines indicate a cutoff value of 1.4. SARS-CoV-2, severe acute respiratory syndrome coronavirus 2.

## Data Availability

The data analyzed during this study are available from the corresponding author on reasonable request.

## Abbreviations

COVID-19: Coronavirus disease
SARS-CoV-2: Severe acute respiratory syndrome coronavirus 2
PCR: Polymerase chain reaction
Ig: Immunoglobulin
Ct: Cycle threshold
