# Supplementary figures and images for: Genetic testing and serological screening for SARS-CoV-2 infection in a COVID-19 outbreak in a nursing facility in Japan
Source: BMC Infect Dis. 2021 Mar 15;21:263. doi: 10.1186/s12879-021-05972-5 (PMC7957465; doi:10.1186/s12879-021-05972-5)

Supplementary Figure 1

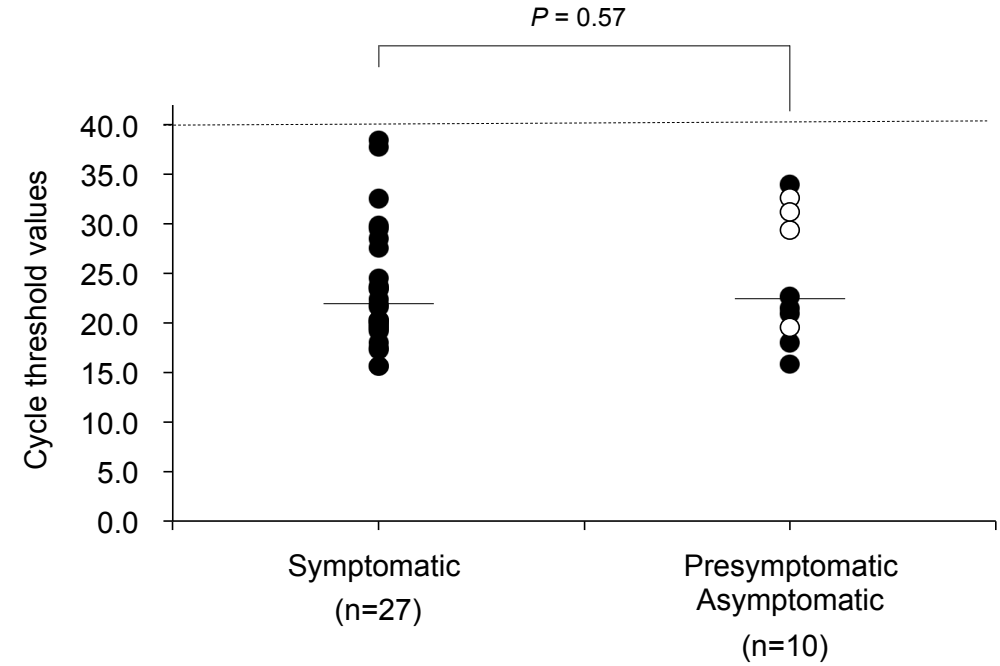

Supplement: Supplementary file 4 — Additional file 4: Supplementary Figure S1. Cycle threshold values for residents and staff with a real-time PCR-positive result for SARS-CoV-2 according to their symptom status. Cycle threshold values for the first positive PCR results of 37 residents and staff were compared between two groups classified based on the symptomatic status at the time of testing (symptomatic vs. presymptomatic/asymptomatic) The symptomatic status is defined in the methods section. The white circles indicate data of individuals with asymptomatic status. The horizontal solid bars indicate the median values. SARS-CoV-2, severe acute respiratory syndrome coronavirus 2 [file 12879_2021_5972_MOESM4_ESM.pdf]

Supplementary Figure 2

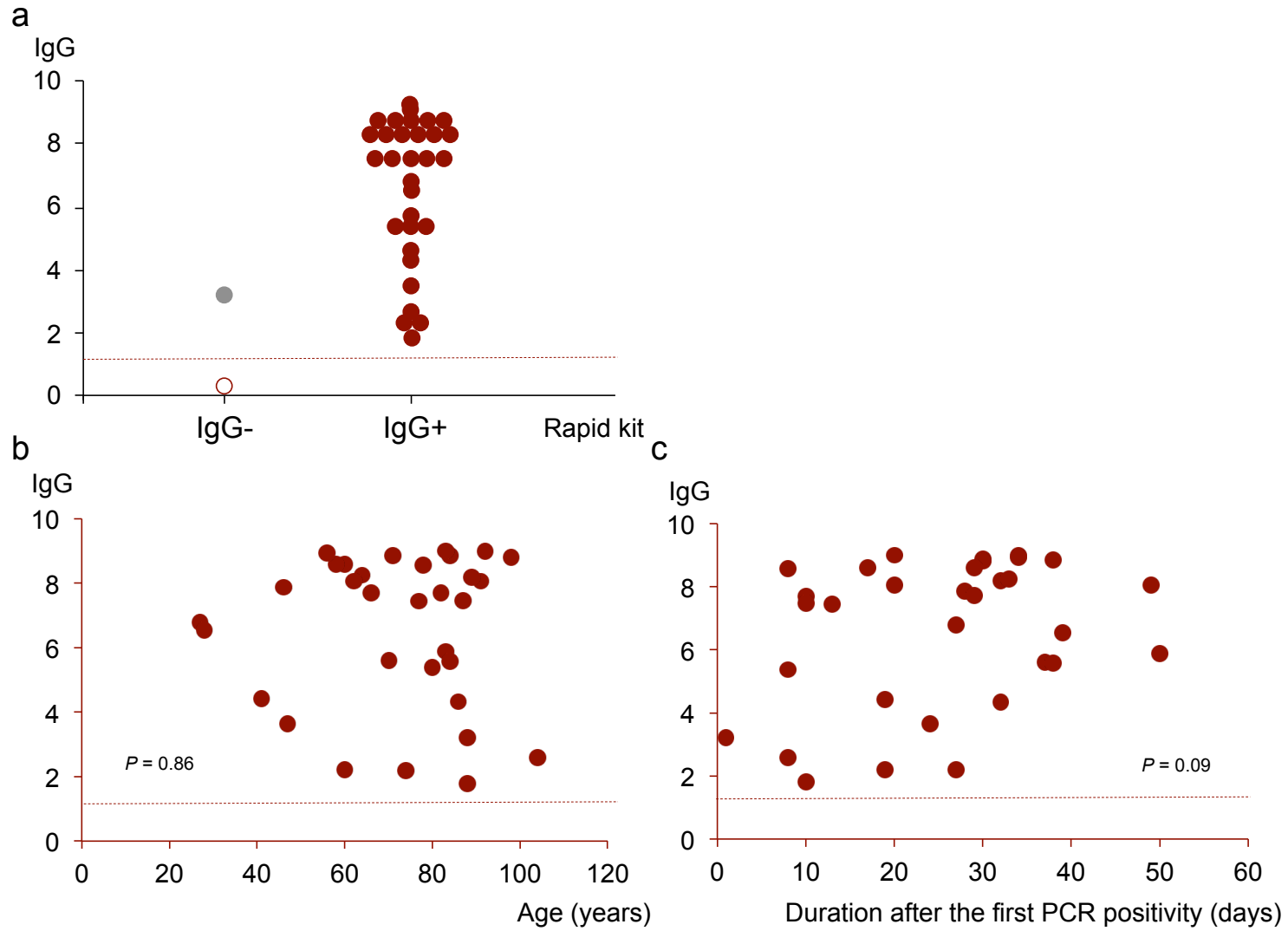

Supplement: Supplementary file 5 — Additional file 5: Supplementary Figure S2. IgG quantification assay in residents and staff with PCR-positive results for SARS-CoV-2. a. Relationship of IgG positivity between rapid kit and quantification tests in 33 PCR-positive residents and staff. The gray circle indicates resident no. 25 from Table S3. The white circle indicates staff no. 10 from Table S3. b, c. Association of age (b) and duration after the first PCR positivity (c) with quantitative IgG values in 32 PCR-positive and quantitative IgG-positive residents and staff. The horizontal dotted lines indicate a cutoff value of 1.4. SARS-CoV-2, severe acute respiratory syndrome coronavirus 2. [file 12879_2021_5972_MOESM5_ESM.pdf]
